# Magnetic Resonance Assessment of Hypertrophic and Pseudo-Hypertrophic Changes in Lower Leg Muscles of Boys with Duchenne Muscular Dystrophy and Their Relationship to Functional Measurements

**DOI:** 10.1371/journal.pone.0128915

**Published:** 2015-06-23

**Authors:** Ravneet S. Vohra, Donovan Lott, Sunita Mathur, Claudia Senesac, Jasjit Deol, Sean Germain, Roxanna Bendixen, Sean C. Forbes, H. Lee Sweeney, Glenn A. Walter, Krista Vandenborne

**Affiliations:** 1 Department of Physical Therapy, University of Florida, Gainesville, FL, United States of America; 2 Department of Occupational Therapy, University of Florida, Gainsville, FL, United States of America; 3 Department of Physiology, School of Medicine, University of Pennsylvania, Philadelphia, PA, United States of America; 4 Department of Physiology and Functional Genomics, University of Florida, Gainsville, FL, United States of America; University of Louisville School of Medicine, UNITED STATES

## Abstract

**Introduction:**

The primary objectives of this study were to evaluate contractile and non-contractile content of lower leg muscles of boys with Duchenne muscular dystrophy (DMD) and determine the relationships between non-contractile content and functional abilities.

**Methods:**

Lower leg muscles of thirty-two boys with DMD and sixteen age matched unaffected controls were imaged. Non-contractile content, contractile cross sectional area and non-contractile cross sectional area of lower leg muscles (tibialis anterior, extensor digitorum longus, peroneal, medial gastrocnemius and soleus) were assessed by magnetic resonance imaging (MRI). Muscle strength, timed functional tests and the Brooke lower extremity score were also assessed.

**Results:**

Non-contractile content of lower leg muscles (peroneal, medial gastrocnemius, and soleus) was significantly greater than control group (p<0.05). Non-contractile content of lower leg muscles correlated with Brooke score (r_s_ = 0.64-0.84) and 30 feet walk (r_s_ = 0.66-0.80). Dorsiflexor (DF) and plantarflexor (PF) specific torque was significantly different between the groups.

**Discussion:**

Overall, non-contractile content of the lower leg muscles was greater in DMD than controls. Furthermore, there was an age dependent increase in contractile content in the medial gastrocnemius of boys with DMD. The findings of this study suggest that T_1_ weighted MR images can be used to monitor disease progression and provide a quantitative estimate of contractile and non-contractile content of tissue in children with DMD.

## Introduction

Muscular dystrophies embrace a large cluster of genetic disorders that result in loss of muscle fiber integrity, leading to progressive skeletal muscle weakness. Duchenne muscular dystrophy (DMD) is one of the most common forms of muscular dystrophy affecting approximately 1 in 3500–6000 newborn males [1, 2]. DMD is caused by a mutation(s) in the *dystrophin* gene leading to the absence or non-functional structural protein, dystrophin (*dys*) [3]. Absence of *dys* protein affects sarcolemmal integrity leading to progressive fatty tissue infiltration and muscle atrophy. Loss of muscle strength and skeletal functional mass in DMD negatively affects the health outcomes leading to an increased risk for disability and morbidity as well as decreased quality of life [4–6]. During the initial stages of the disease, inflammatory changes and repair are seen which are followed by fatty tissue infiltration [7]. This progressive fatty tissue infiltration and muscle weakness leads to loss of ambulation between the ages of 8–12 years [8, 9]. Disease progression ultimately leads to premature death due to cardiopulmonary complications in the early twenties [10].

Mounting evidence suggests that effects of dystrophin deficiency vary among species, individuals and muscles. Hypertrophic and atrophic changes have not only been documented in children with DMD but also in the preclinical models of DMD. For example, in the *mdx* mouse model diaphragm muscle is affected much earlier in life compared to the peripheral skeletal muscles [11]. Furthermore, skeletal muscles of *mdx* mice exhibit hypertrophic changes between 10–40 weeks of age followed by skeletal muscle atrophy during later life [12, 13]. On the other hand, the feline model of DMD displays persistent muscle hypertrophy throughout their life span [14, 15]. In contrast, the golden retriever model of muscular dystrophy (GRMD) demonstrates skeletal muscle atrophy and is believed to most closely resemble the pathophysiology of patients with DMD [16, 17]. Selective muscle involvement is also seen in boys with DMD, which is evident from atrophy, hypertrophy, and even sparing of different limb skeletal muscles. For example, in DMD patients there is a significant loss of skeletal muscle mass of the pelvis and thigh muscles [18–21]. Alternatively, studies have suggested selective sparing as well as hypertrophy of gracillis (Gr), sartorius (Sar), semimembranosus (SM) [18, 19], and tibilais posterior (TP) muscles [22, 23]. Moreover, leg muscles, especially, the ankle plantar flexors demonstrate an enlarged appearance commonly termed as ‘pseudo-hypertrophy’. Pseudo-hypertrophy, a hallmark sign of DMD is caused by replacement or infiltration of muscles by fatty and/or collagenous tissue. Pseudo-hypertrophy has been reported in boys with DMD in the gastrocnemius, infraspinatus, deltoid, and temporalis muscles [24, 25]. Along with pseudo hypertrophic changes, hypertrophy has also been reported in gastrocnemius, gracillis and sartorius [19, 26]. Collectively, these studies show that some muscles are undergoing both hypertrophic and pseudo hypertrophic changes at the same time in DMD. In order to measure the size and composition of the muscles, various invasive and non-invasive techniques have been incorporated.

Muscle biopsy has been regarded as a gold standard to assess the muscle involvement in patients with neuromuscular diseases. However, it becomes impractical to perform repeated muscle biopsies on children with DMD. Also, since DMD has selective muscle involvement, biopsy of a single muscle may fail to provide an accurate assessment of the disease progression. Therefore, there is a dire need for a sensitive, non-invasive biomarker in order to effectively monitor the disease progression as well as evaluate the potential therapeutic interventions in boys with DMD. A variety of imaging techniques, such as CT and ultrasound, have been used to monitor the disease progression in neuromuscular disorders [27–29]. On the other hand, developments in advanced magnetic resonance imaging (MRI) techniques have encouraged investigators to explore the potential of advanced techniques for better spatial and contrast resolution. MRI has been used to monitor alterations in skeletal muscles in conditions like obesity [30], sarcopenia [31], cachexia [32] and spinal cord injury [33, 34]. Furthermore, MRI has also been used to monitor disease progression in various myopathies [35, 36]. An increased body of evidence suggests that MRI may have the potential to be a sensitive biomarker of muscle involvement in DMD. MRI methods, such as T_1_- and T_2_- weighted imaging as well as three point DIXON imaging, have been utilized to estimate intramuscular fatty infiltration in patients with DMD [21, 35, 37, 38]. Recently, our group demonstrated age related changes in cross sectional area (CSA) of lower extremity muscles and specific torque production in boys with DMD [20]. Assessing muscle CSA is an important measure in muscular dystrophy patients because it provides information about changes in size of the muscles [19, 20]. However, quantifying CSA fails to provide vital information regarding the contractile and non-contractile content of a muscle, which may reveal additional, and more clinically useful information pertaining to the muscle quality [37]. Indeed, our group and others have estimated the contractile and non-contractile content in thigh muscles of boys with DMD [18, 37, 39]. On the contrary, there are only a few studies that have quantified the amount of contractile and non-contractile tissue in lower leg muscles [23, 40]. The reason for paucity of such studies may be the relatively slower rate of disease progression in distal leg muscles compared to the proximal muscles. However, examination of lower leg muscles may also be advantageous as muscle involvement is slower, thereby providing a greater range of ages to test the potential therapeutic interventions.

Therefore, the purpose of this study was to assess contractile and non-contractile content of individual lower leg muscles. Specifically, the aims included: 1) to assess the contractile and non-contractile content of individual leg muscles in boys with DMD and age matched healthy controls; 2) to evaluate age related changes in contractile and non-contractile content of lower leg muscles in boys with DMD; and 3) to evaluate the relationship between non-contractile content and functional ability of boys with DMD

## Research Design and Methods

### Ethics Statement

Ethical approval was obtained from the Institutional Review Board (IRB) at the University of Florida (UF). The study was in compliance with the Health Insurance Portability and Accountability Act (HIPAA). A parent/guardian of each subject was required to sign the consent form approved by the IRB. Subjects were also required to provide signed assent to participate.

### Participants

A cohort of thirty-two boys with DMD group (DMD) and sixteen healthy boys from the general population (Ctrl) volunteered to participate in this observational cross-sectional study. Ambulatory status and physical characteristics of all subjects are shown in Table 1. A report confirming the diagnosis of DMD using molecular genetic testing (e.g. PCR amplification) and/or immunohistochemical staining from muscle biopsy was obtained from each of the DMD subjects. In the DMD group, twenty-nine boys were ambulatory and three boys were non-ambulatory. All DMD subjects were being treated with corticosteroids (either Prednisone or Deflazacort). Ctrl subjects were relatively sedentary, in that they did not participate in sport specific training 2 or more times per week.

**Table 1:** Subject Demographics of Duchenne muscular dystrophy (DMD) and age matched control (Ctrl) boys.

Age group	Ctrl/DMD	Age (years)	Height (m)	Weight (kg)	BMI (kg/m^2^)	BSA (m^2^)
5–7.9 years	Ctrl (n = 5)	6.68 (0.61)	1.21 (0.03)	22.70 (2.45)	15.38 (0.99)	0.87 (0.06)
	DMD (n = 13)	6.51 (0.82)	1.13 (0.09) **	21.63 (2.40)	16.89 (1.78)	0.73 (0.05)
8–9.9 years	Ctrl (n = 4)	8.67 (0.52)	1.43 (0.07)	39.45 (8.09)	19.26 (4.01)	1.25 (0.14)
	DMD (n = 12)	9.01 (0.54)	1.26 (0.07)**	30.37 (8.23)**	19.00 (3.60)	0.86 (0.09)
> 10 years	Ctrl (n = 7)	12.31 (1.85)	1.56 (0.09)	42.33 (8.27)	17.37 (2.75)	1.35 (0.17)
	DMD (n = 7)	12.51 (1.89)	1.34 (0.06)**	44.14 (8.41)	24.47 (3.81) **	1.28 (0.15)

** p<0.05 versus Ctrl group within age group.

Values are mean (SD); Body mass index (BMI); Body surface area (BSA)

### MRI acquisition

MRI was performed on a 3.0T (Achieva, Phillip Medical System) whole-body scanner. Subjects were placed in a supine position with their lower leg positioned in an 8-channel sensitivity encoding, receive-only extremity coil (3.0T) for lower leg imaging. Three-dimensional trans-axial gradient echo images without fat suppression were acquired with the following parameters: field of view (FOV) 120 X 120 X 146 mm^3^, repetition time (TR) 4.9 ms, echo time (TE) 1.9 ms, flip angle 20°, and slice thickness 5.6 mm.

### Isometric muscle strength testing

Isometric peak torque of ankle plantar flexors (PF), and dorsiflexors (DF) of the right leg was measured using a Biodex dynamometer. For PF and DF, the knee was placed between 0° to 10° of flexion and the ankle was placed in a neutral position (e.g. the talocrural joint flexed at 90° angle, for PFs) or 30° of plantar-flexion (for DFs). The subject was instructed to push or pull as hard as possible for 5 seconds, followed by a 1–2 minute rest. Five trials were performed for each muscle group, and the highest torque value was used for analysis (peak torque). This protocol was tested for between-day reliability (2-month interval) by the same tester, in a subset of children with DMD (n = 6) and controls (n = 10). ICCs were calculated for both groups of subjects. High reliability was found for PFs: ICC = 0.88 in DMD and 0.98 in controls, and DFs: ICC = 0.87 in DMD and 0.95 in controls; p = .001 for all ICCs).

### Functional abilities

Subjects were asked to perform timed functional tasks. These tasks included the time to walk 30 feet (30 feet Walk), time to rise from floor (Supine up), time to rise from a chair (Chair Up), the pediatric timed up and go (TUG) and ascend four stairs (4 stairs) [41]. Subjects were asked to perform each test three times, and the fastest time was recorded for analysis. The functional ability was also ranked using the Brooke Lower Extremity Functional scale [42]. This scale is ranged from grade 1 (able to walk and climb stairs independently) to grade 10 (confined to bed).

### MRI data analysis

**Fig 1:**
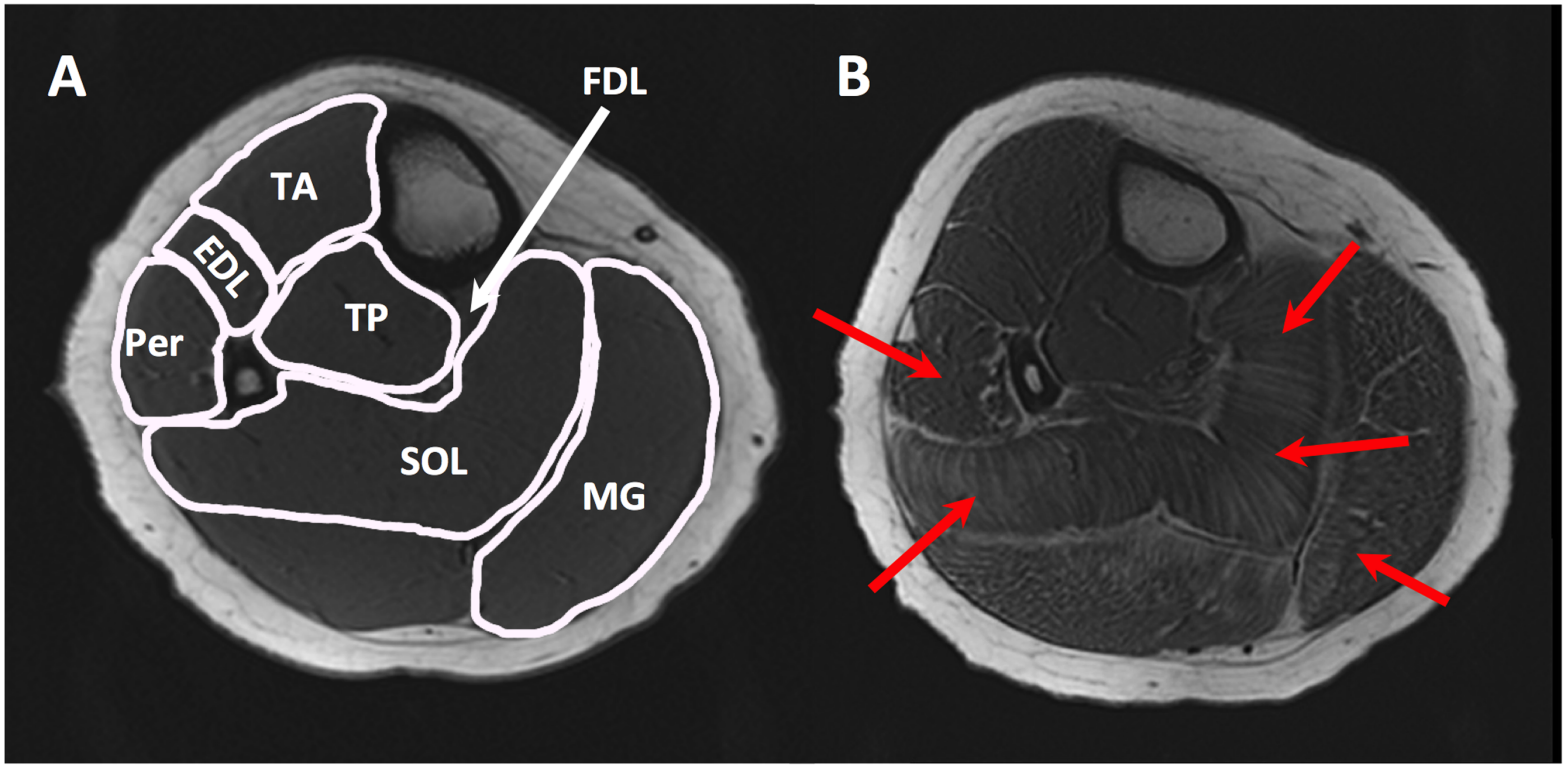
T_1_ weighted images of lower leg muscles. Representative T_1_ weighted 3-D gradient echo images of A) control, and B) DMD subject. Outline of regions of interest (A) showing different muscles namely TA, tibialis anterior; EDL, extensor digitorum longus; Per, peroneal; MG, medial gastrocnemius; SOL, soleus; TP, tibilais posterior; FDL, flexor digitorum longus. (B) Showing areas of fatty tissue infiltration in leg muscles.

Medical Image Processing, Analysis and Visualization (MIPAV) software (version 4.2.1; National Institutes of Health, MD) was used to analyze the MR images. We selected a single image that corresponded to the most proximal slice in which the flexor digitorum longus (FDL) could be visually confirmed. Furthermore, to improve the coverage and reliability we selected the subsequent proximal and distal slices for data analysis (three slices total per subject). We identified 5 individual muscles on the image: tibialis anterior (TA), extensor digitorum longus (EDL), peroneal (PER), medial gastrocnemius (MG) and soleus (SOL) (Fig 1).

Contractile cross sectional area (C-CSA), non-contractile cross sectional area (NC-CSA), and non-contractile content (%) (NCC) in the lower leg muscles were calculated using a similar image analysis technique as previously described [31, 43]. The first step of the analysis was to correct for image heterogeneity caused by sub-optimal radiofrequency coil uniformity, or gradient-driven eddy currents, using a well-established nonparametric non-uniform intensity normalization (N3) algorithm [44]. This step was essential for subsequent analyses that assume homogenous signal intensity across the images. Optimized image correction parameters (N3) were determined (end tolerance 0.0001; maximum iterations 100, signal threshold 1; field distance 33.33 mm; subsampling factor 2; Kernel full width half maximum of 0.15; Wiener filter noise 0.01), and the same parameters were applied to all images.

A region of interest (ROI) was drawn by manually tracing the tibialis posterior (TP) muscle and subcutaneous fat. TP was chosen as the reference muscle as it has been shown to be relatively spared in DMD subjects [23]. The total number of pixels, histogram of all the pixels and signal intensities within the ROIs were produced. To separate contractile and non-contractile tissues in the pixel number-signal intensity histogram with minimal bias, we implemented the Maximum Entropy method [37]. Furthermore, in order to minimize manual tracing errors, three trials were averaged, and the values were applied to all muscles of interest for each slice of interest. After tracing all muscles of interest, the following parameters were computed: 1) total number of pixels with in ROI; 2) the number of pixels with signal intensity lower than the threshold value (contractile tissue); and 3) the number of pixels with a value higher than the threshold value (non-contractile tissue). The proportion of contractile and non-contractile content of each muscle was calculated as described previously [37]. Intra class correlation coefficient in individual muscles between analyzers ranged from 0.93 to 1.00 for non-contractile content.

### Statistical analysis

Statistical analysis was performed using Graph Pad prism version 6.0. Data were described using means and standard deviations (SD). A Non-parametric (Mann Whitney *U*-test) was performed to compare non-contractile content, contractile CSA and non-contractile CSA between DMD and Ctrl groups. Within-group comparisons were made using Wilcoxon (two related sample) test and Bonferroni correction was used for multiple comparisons. The significance (two-tailed) values (*p*) were reported for all the comparisons. For the functional tests, the subjects with DMD who were physically unable to perform a test were given the highest score in that activity. Therefore, Spearman’s rank correlation test was used to determine the relationships between two independent variables. The level of significance was set at p ≤ 0.05.

## Results

### Subject demographics

Boys with DMD were shorter in height across all age groups and boys over 10 years of age had higher body mass index compared to controls (Table 1). No significant differences were found in age and body surface area (BSA) between DMD and Ctrl groups. Furthermore, the mean Brooke score was 2.2±0.4 in DMD boys indicating that most patients in this group were ambulatory, except for three subjects who were non-ambulatory.

### Comparison of lower leg muscles of control and DMD group

**Fig 2:**
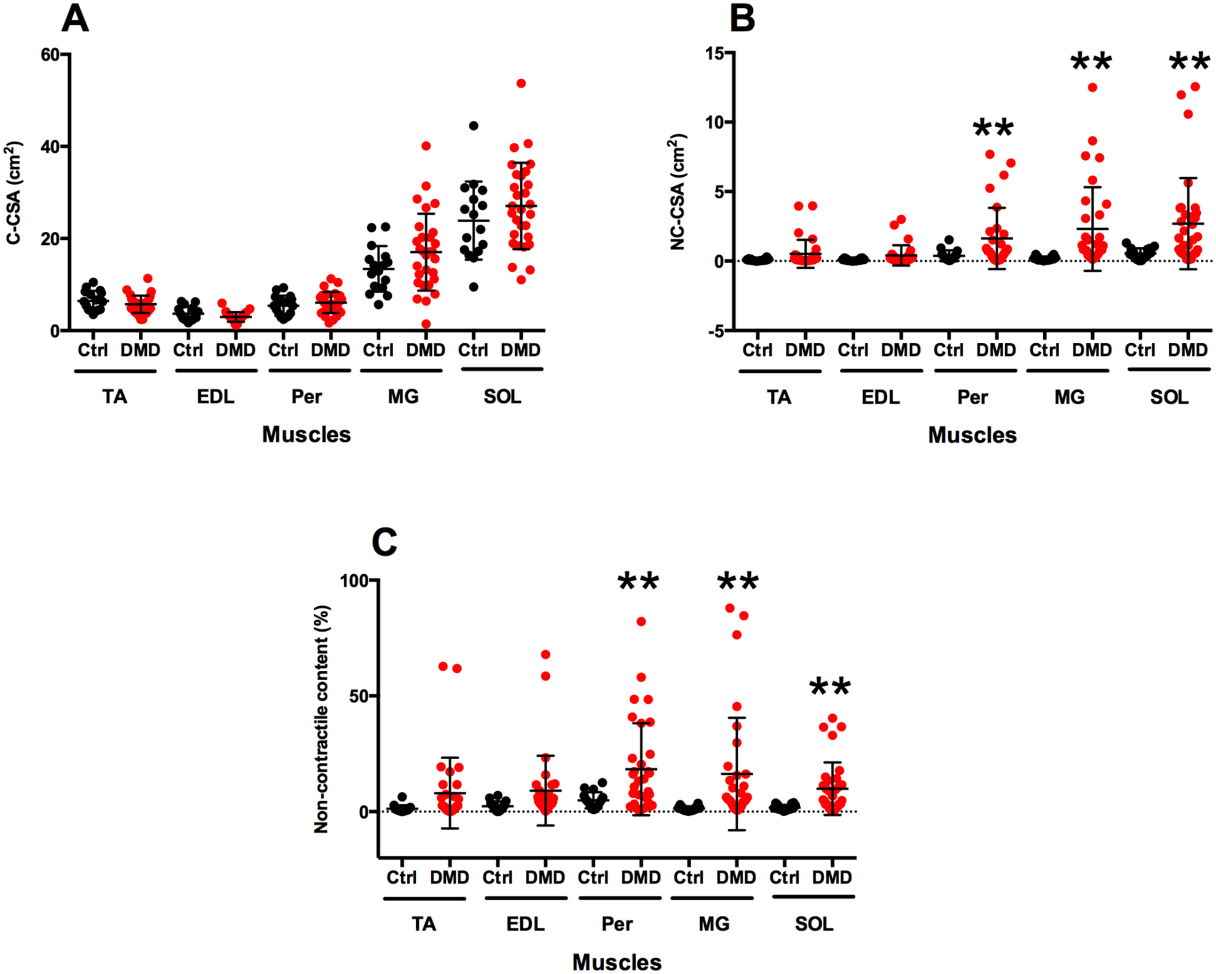
Contractile cross sectional area (C-CSA), non-contractile cross sectional area (NC-CSA), and non-contractile content (NCC) of all the lower leg muscles of boys with DMD and healthy control subjects. Significant difference was found in NC-CSA and of peroneal (Per), medial gastrocnemius (MG), and soleus (SOL) (denoted by **, p<0.05). No differences were observed in C-CSA of DMD and Ctrl leg muscles when all age groups were combined. Data is presented as mean (SD).

Using MR T_1_ weighted images, considerable differences in leg muscles were observed in the DMD group. C-CSA (cm^2^), NC-CSA (cm^2^), and NCC (%) of lower leg muscles of the DMD and Ctrl groups were calculated. We did not observe any significant difference in C-CSA between the DMD and Ctrl groups when all age groups were combined (Fig 2A). However, NC-CSA of Per, MG and SOL differed significantly between the DMD and Ctrl groups (Fig 2B). Additionally, we found that NCC of Per, MG and SOL in the DMD group differed significantly from the Ctrl group (Fig 2C). On the other hand, C-CSA, N-CSA and NCC of TA and EDL did not differ significantly between the DMD and Ctrl groups (Fig 2).

### Muscle non-contractile content in control and DMD in each age group

**Fig 3:**
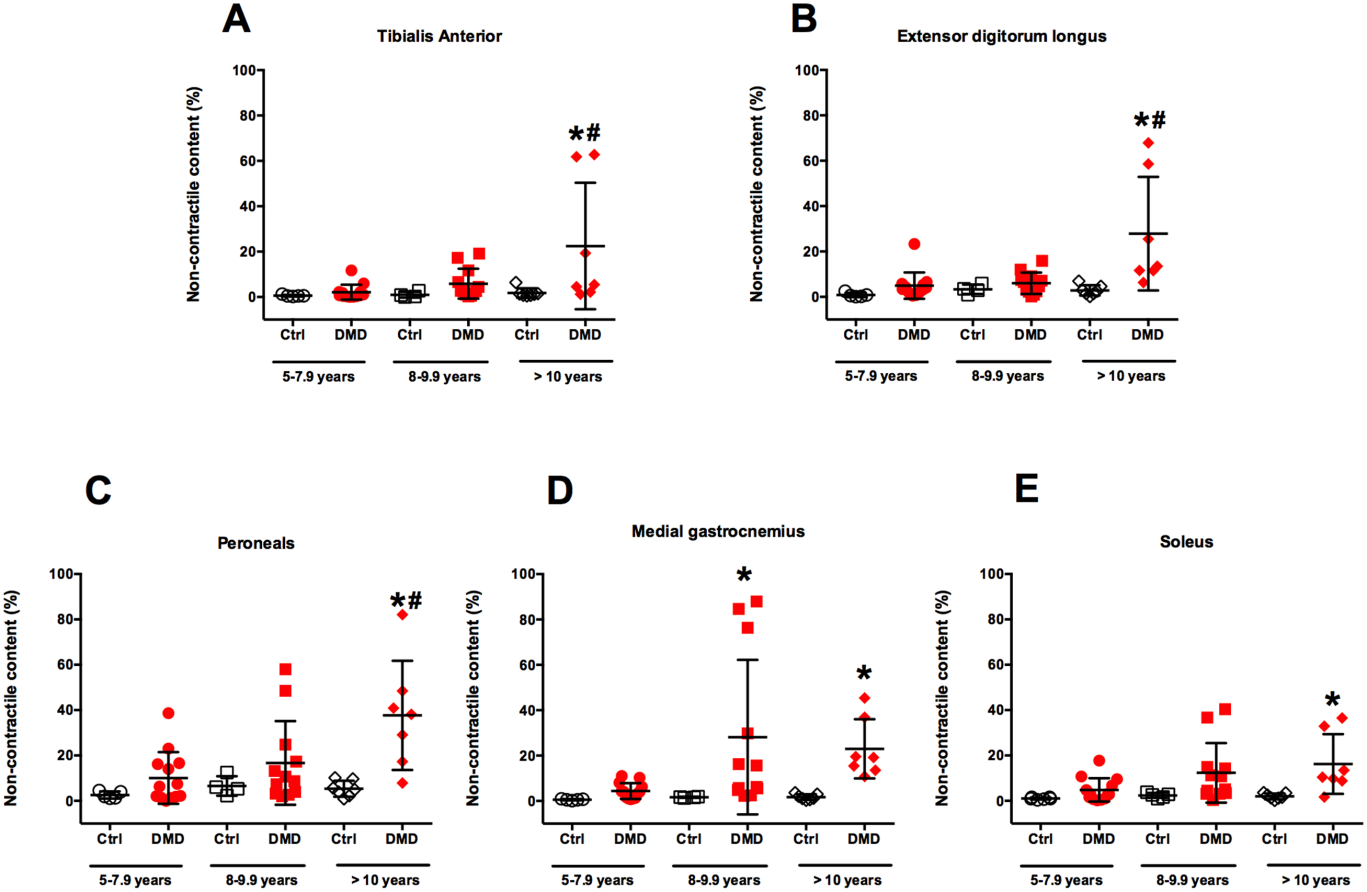
Percent non-contractile content (NCC) in leg muscles of Ctrl and DMD in different age groups. NCC of leg muscles of DMD at the age of >10 years was significantly different from 5–7.9 and 8–9.9 years age groups (denoted by #, p<0.05). NCC of TA, EDL, Per, MG, and SOL of DMD were significantly different than Ctrl in > 10 year age group (denoted by *, p<0.05). Additionally, only MG showed significant differences between DMD and Ctrl group at the age of 8–9.9 year. Data is presented as mean (SD).

To examine age-related changes in NCC, the participants (Ctrl and DMD) were divided into three age groups (5–7.9 years, 8–9.9 years, > 10 years; see Fig 3). Overall, in the Ctrl group, we did not observe an age dependent increase in NCC of the leg muscles. However, significant differences were found in the muscles of the DMD group. Specifically, the dorsiflexor muscles (TA and EDL) had a greater NCC in DMD compared with Ctrl in the oldest age group (> 10 years) only. Furthermore, except for MG, we found that NCC of plantar flexor muscles in the oldest DMD group (> 10 years) was significantly greater than younger age groups (5–7.9 years and 8–9.9 years). Surprisingly, we observed a significant increase in NCC of MG in 8–9.9 years old DMD group compared to age matched Ctrl group. Collectively, these results indicate that there is age dependent increase in NCC of lower leg muscles in boys with DMD (Fig 3).

### Age related changes in contractile and non-contractile CSA

**Fig 4:**
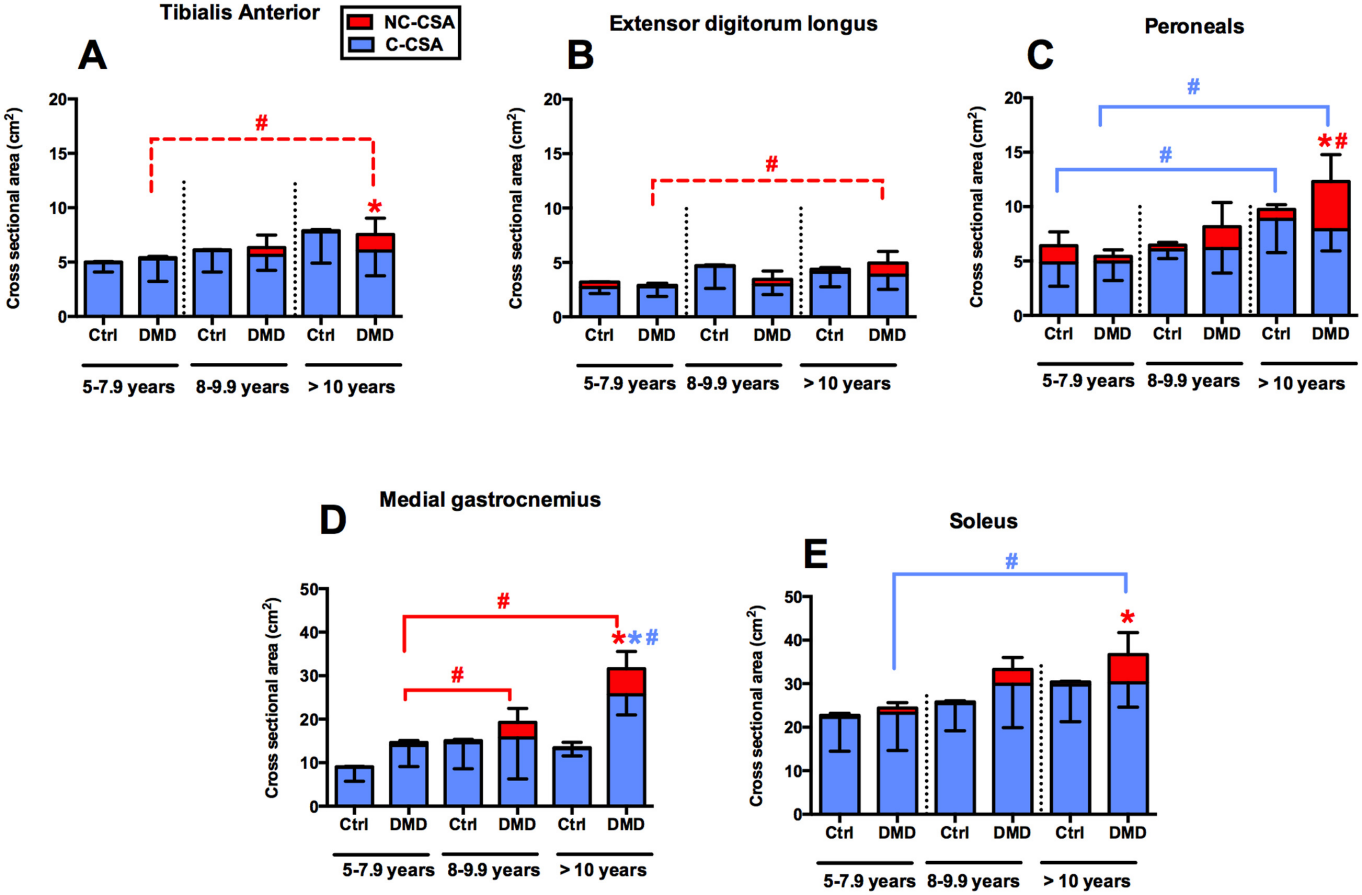
Comparison of C-CSA and NC-CSA among different age groups in DMD and control groups. Red and blue color represents N-CSA and C-CSA respectively. Symbol colors are accordant with the bar colors (red; N-CSA, blue; C-CSA). * Represents significant differences between DMD and age matched Ctrl group (p<0.05); # represents significant differences across the age groups (p<0.05).

Muscles of the lower leg were evaluated for age related changes in C-CSA and NC-CSA (Fig 4). Overall, there was an age dependent progressive increase in both C-CSA and NC-CSA. Boys with DMD who were > 10 years old displayed a significant increase in NC-CSA in all the muscles compared to the youngest boys with DMD (5–7.9 years). Additionally, between 8–9.9 and >10 years, NC-CSA was significantly different in PER and MG (p<0.001). On the other hand, C-CSA of PER and MG and SOL differed significantly between the youngest (5–7.9 years) and the oldest group (>10 years) (p<0.01). Finally, only the MG displayed significant increase in contractile area compared to age matched Ctrl in the oldest group (> 10 years).

### Relationships between non-contractile content of lower leg muscles and functional abilities

**Fig 5:**
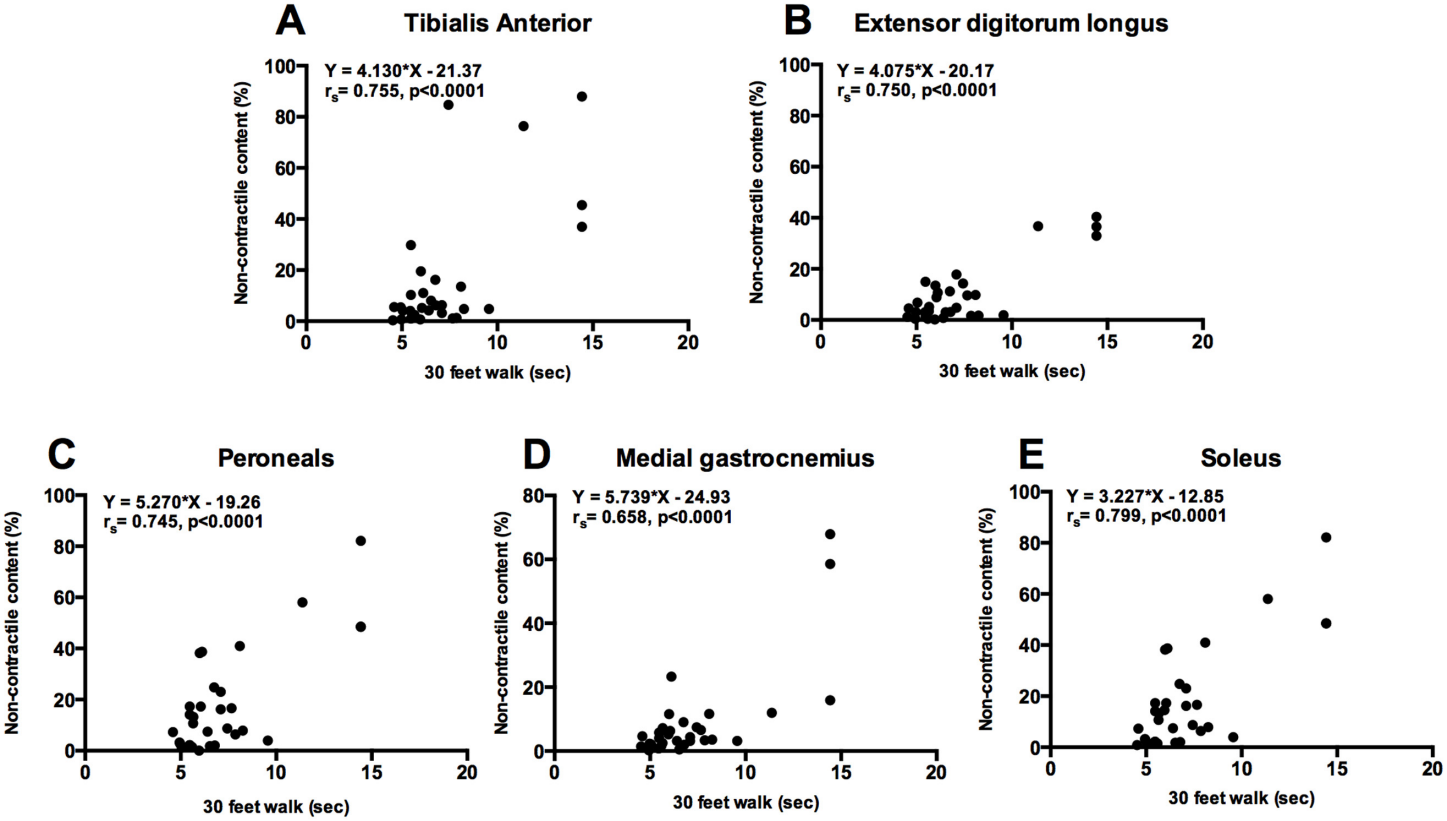
Relationship between NCC of different leg muscles and 30 feet walk (sec). There was a significant relationship between increase in NCC of all leg muscles and time taken to cover 30 feet distance (r_s_; 0.65–0.80, p<0.0001).

**Fig 6:**
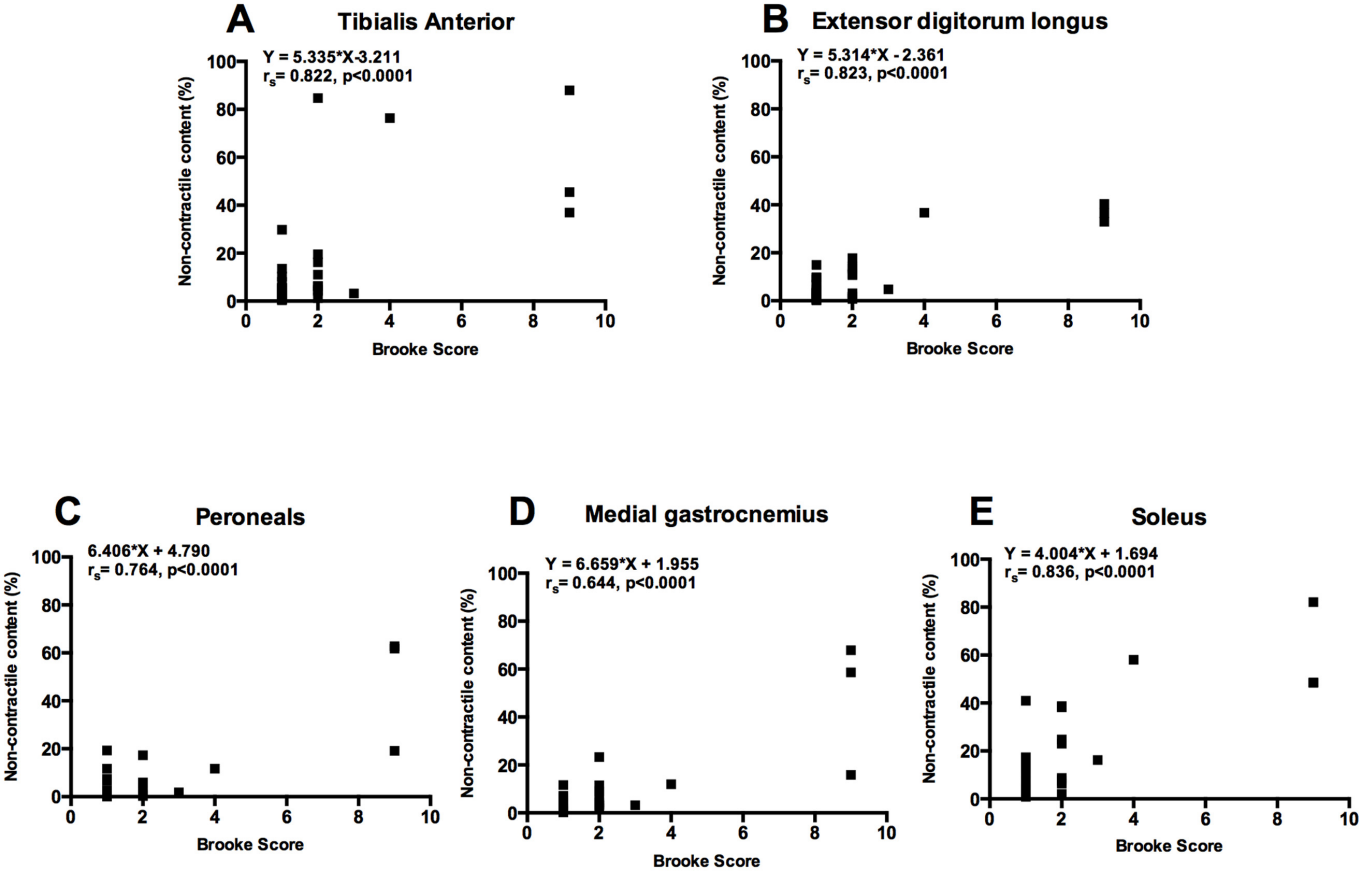
Relationship between NCC of different leg muscles and Brooke score. There was a significant relationship between NCC of all leg muscles and Brooke score (r_s_; 0.64–0.84, p<0.0001).

**Fig 7:**
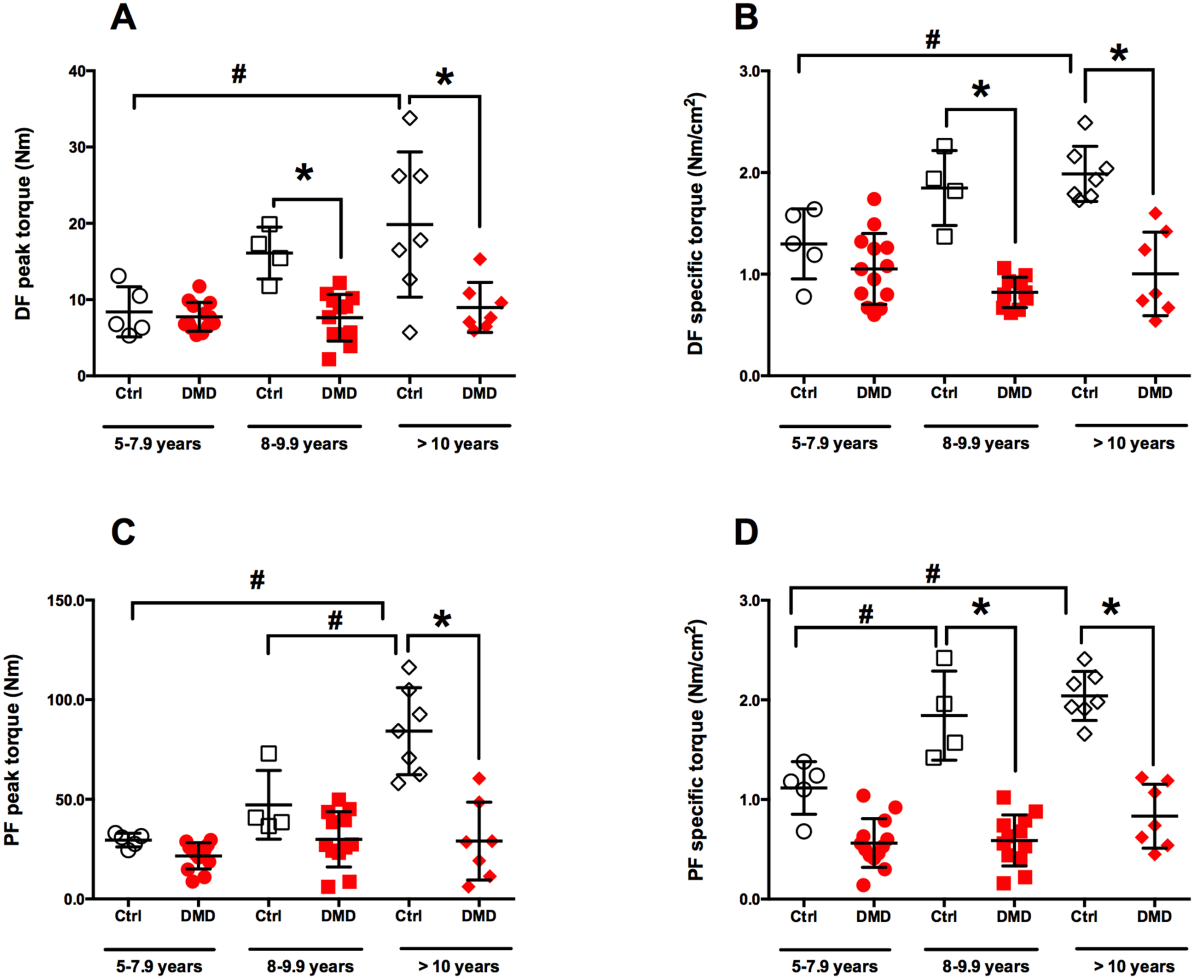
Comparison of dorsiflexors (DF) and plantarflexors (PF) peak torque (A, B) and specific torque in boys with DMD and healthy Ctrl across different age groups. * represents significant differences between DMD and Ctrl within age groups (p<0.05), # represents significant differences across the age groups (p<0.05). Data is presented as mean (SD).

The relationships between the NCC in muscle groups, strength measurements, timed functional tests and Brooke score were examined in DMD subjects (Table 2). (A) Significant correlations were found in NCC of all muscles and the 30-feet walk (r_s_ = 0.66–0.80, p<0.0001) (Fig 5) and Brooke score (r_s_ = 0.64–0.84, p<0.0001) (Fig 6). (B) In addition, we found that both peak torque and specific torque of DF and PF were significantly reduced in DMD as compared to Ctrl group. As expected, there was a significant increase in DF and PF force production with age in the Ctrl group but the DMD group did not show any increase in force production with age (Fig 7).

**Table 2:** Comparison of timed functional tests between control (Ctrl) and subjects with Duchenne muscular dystrophy (DMD).

Timed Functional measurements	30- feet walk	Fastest supine up	Chair up	TUG	4 stairs
**Ctrl**	4.7 ± 0.53	1.6 ± 0.27	0.6 ± 0.18	5.9 ± 0.87	1.8 ± 0.44
**DMD**	6.48 ± 1.53**	4.33 ± 2.47**	0.88 ± 0.41	6.76 ± 1.21	3.73 ±1.42**

**p<0.05 versus Ctrl group.

Values are mean (SD).

## Discussion

Muscle MRI is being increasingly used as a diagnostic tool in various inherited neuromuscular disorders [20, 21, 38]. In DMD, T_1_ weighted images have been used to demonstrate heterogeneity in fatty tissue infiltration among the lower limb muscles in older boys [45]. However, there is a dearth of studies quantifying the fatty tissue infiltration in the lower leg muscles. In this study we provided a quantitative estimate of fatty tissue accumulation in the lower leg skeletal muscles of DMD and unaffected controls using T_1_ weighted MR images. The main findings of this study were: (1) boys with DMD had significant increased proportion of NCC and NC-CSA in the PER, MG and SOL compared to age matched controls; (2) DMD boys had a relatively greater increase in NC-CSA with age compared to C-CSA; (3) MG in older DMD boys exhibited greater contractile area as compared to age matched controls; (4) DF and PF specific torque production were significantly reduced in DMD boys as compared to healthy control boys; and (5) the proportion of NCC of leg muscles correlated with functional ability as assessed by 30 feet walk and Brooke score, and muscle strength measurements from dynamometer muscle testing.

### Variable muscle involvement in DMD

There is an increasing body of evidence showing progressive and selective pattern of involvement in muscles of DMD patients [18, 19]. Accordingly, our results generally support the findings of previously published results and confirm that there is indeed a considerable amount of variation in the rate of disease progression across the lower leg muscles. While a significant increase in plantar-flexor NCC was seen, NCC of dorsiflexor muscles (TA and EDL) did not differ significantly from the Ctrl group, especially in the younger age groups. Previous cross sectional investigations in boys with DMD have reported that the TA and EDL muscles are relatively spared as compared to other muscles [26, 29]. The etiology for this selective muscle involvement and relative sparing of lower leg muscles is still unclear. One potential consideration is that the muscles that are relatively spared may undergo reduced loading during eccentric contractions with daily activities such as walking. Indeed, recent studies using quantitative gait assessment have reported altered gait patterns in DMD children [46–48]. Furthermore, the study by Voloshina et al. (2013) testing effects of walking on uneven terrain in healthy subjects, reported increased EMG activity of the SOL and MG by 28% and 17% respectively whereas the TA did not exhibit significant changes in the mean activity. The results from our study further support the concept that dorsiflexor muscles are relatively spared in DMD especially in the younger age group.

### Age related changes in fatty tissue infiltration in dystrophic muscles

Dystrophic skeletal muscles are characterized by repeated cycles of degeneration and regeneration, which eventually lead to replacement of viable contractile muscle tissue with non-contractile tissue. In this study, using MRI-T_1_ weighted images we were able to quantify fatty tissue infiltration in lower leg muscles of boys with DMD and confirmed an age dependent increase in NCC and NC-CSA of Per, MG and SOL muscles. Interestingly, up until 10 years of age the TA and EDL appeared to be relatively preserved. However, there was a significant increase in NCC of the TA and EDL especially in the older group of DMD children (> 10 years) compared to younger DMD subjects. Overall, the extent of fatty tissue infiltration in all the leg muscles of boys with DMD examined in this study was significantly different between the youngest (5–7.9 years) and the oldest age group (> 10 years). These results are consistent with the previous studies documenting an increase in fatty tissue infiltration in the dorsiflexor as well as plantar flexor muscles of DMD boys [38, 49]. For example, Forbes et al. (2014) reported age dependent increases in MRI-T_2_ of the TA, Per, MG and SOL muscles and this increase was associated with increased lipid accumulation. Furthermore, the findings in our study are in accordance with the previous studies in which the authors reported 18–38% in fatty-fibrous tissue in the gastrocnemius muscle whereas this component did not exceed 8% in unaffected subjects [50]. Intriguingly, results of our study revealed that the MG was the only muscle that had a significant increase in NC-CSA at the age of 8–9.9 years, which is in contrast with previously published findings [40]. The study by Beenakker et al. (2002) observed an increase in calf circumference in boys with DMD aged 4–8 years. However, we did not find any significant difference in NC-CSA of MG in 5–7.9 year group. The discrepancy with the latter study could be due to the differences in the methodology that was incorporated. The Beenakker et al. (2002) study quantified calf enlargement using a tape measure, which fails to distinguish between contractile and non-contractile component of muscles.

### Pseudo hypertrophic and hypertrophic changes in dystrophic muscles

The composition of enlarged calf muscles in DMD is still debated [26, 50]. We used an objective MR measure to provide a quantitative estimate of contractile and non-contractile content in leg muscles of DMD boys. Our results indicate that there was an age dependent increase in C-CSA and NC-CSA of plantar flexor muscles in DMD boys. Specifically, we found that, NC-CSA of PER (DMD, 79% greater than Ctrl), MG (DMD, 92% greater than Ctrl) and SOL (DMD, 82% greater than Ctrl) were significantly greater in DMD than Ctrl in the oldest age group (>10 years) suggesting pseudo hypertrophic changes in the plantar flexor muscles of DMD boys. A similar pattern of muscle involvement has been previously reported in DMD patients [23, 51]. Although the NC-CSA increased significantly with age in PER, MG and SOL muscles, TA and EDL seemed to be relatively spared especially until the age of 10 years. Similarly, recent studies [22, 23, 49] have shown that the TA is a relatively preserved muscle especially during the early stages of life. Furthermore, in the present study we reported a significant increase in C-CSA of PER and MG (38% and 45% respectively) in the oldest age group (>10 years) compared to youngest (5–7.9 years) group. Interestingly, we found that there was almost a 45% increase in the MG C-CSA of DMD boys compared to controls in the oldest age group (>10 years). Collectively, these results suggest that apart from fatty tissue infiltration there are ongoing hypertrophic changes, especially in the MG. Similar to our results, previous studies using histological measures as well as ultrasound have reported true hypertrophy in the calf muscles of DMD boys [26, 50, 52]. Currently there is no cure for DMD, however during the past decade there has been increased demand for novel therapeutic interventions that have the potential to improve the quality of life of DMD patients. It has been suggested that interventions for DMD boys may be most effective during the early stages of disease progression when they have not experienced significant muscle deterioration. Our results provide supporting evidence to this thought as all the leg muscles of DMD boys examined in this study, have significantly less fatty tissue infiltration in the youngest age group (i.e. 5–7.9 years) compared to the older age groups. Therefore, therapeutic interventions aiming to improve muscle quality should be initiated before or during this age while there is viable muscle tissue.

### Muscle contractile area and functional abilities

The amount of force generated per unit muscle mass (specific force) is an important determinant of muscle quality. Our study indicated that both DF and PF peak torque as well as specific torque was lower in DMD than controls, especially in the age groups of 8–9.9 years and > 10 years. Somewhat surprisingly, we did not observe an age dependent decline in peak torque nor specific torque in leg muscles of DMD boys. In fact the specific torque remained relatively constant across the age groups for the boys with DMD. The finding of less specific torque in DMD compared to controls could be attributed to impaired motor unit recruitment in muscles of DMD boys. Previous work supports this concept as a decreased specific tension in both animal models [53, 54] and patients with myotonic dystrophy [55] have been reported. Furthermore, our results and others [20, 23] indicate relative sparing of ankle dorsiflexor muscles in DMD, especially during the early stages of the disease progression. Future studies are warranted to confirm impaired motor unit recruitment in DMD boys and the potential mechanism for this impairment.

### Limitations

There were some limitations to our study. First, even after correcting the images with N3 inhomogeneity correction we were unable to do so completely. We did not include the lateral gastrocnemius because of the presence of B1 inhomogeneity especially in this region. Second, because of limited spatial resolution MR images suffer from partial volume filling possibly leading to underestimation of the contractile area. Moreover, studies have reported that the proportion of non-contractile tissue is not uniformly distributed along the entire length of the muscles and the proportion of non-contractile tissue is greater near origin and insertion of the muscles [56]. Future studies would benefit from carrying out longitudinal studies incorporating larger cohorts of DMD patients and estimating contractile and non-contractile content along the length of the muscle, which can be accomplished by using MRI. Furthermore, this method was not able to account for fibrosis. Fibrotic tissue is expected to have low signal intensity with the T_1_ weighted images acquired, and therefore these pixels may be incorporated with the contractile component of the analysis, resulting in a potential small amount of error when calculating contractile tissue. Lastly, due to a small sample size we were not able to investigate the effects of corticosteroids on the fatty tissue infiltration in the lower leg muscles.

## Conclusions

Overall, this study supports the fact that T_1_ weighted images may be sensitive to disease progression and therefore may be used to test the efficacy of potential therapeutic interventions. Furthermore, MR imaging techniques have the advantage of being less dependent on motivation and coordination, which are major contributors to the variability associated with the functional tests [57]. Additionally, this study supports the notion of progressive and selective involvement and progression of change in the leg muscles of boys with DMD. Finally, we have shown that there is an increase in C-CSA of both the PER and MG with age which may suggest “compensatory hypertrophy”. Therefore, it is important to consider the age as well as target muscle group while testing the novel therapeutic interventions in this patient population.
